# Response of the authors regarding article “Electrocardiographic markers of increased risk of sudden cardiac death in patients with COVID‐19 pneumonia”

**DOI:** 10.1111/anec.12870

**Published:** 2021-07-21

**Authors:** Mohammed Alareedh, Hussein Nafakhi, Foaad Shaghee, Ahmed Nafakhi

**Affiliations:** ^1^ Internal Medicine Department Medicine College University of Kufa Najaf Iraq; ^2^ Internal Medicine Department Jabir ibn Hayyan Medical University Faculty of Medicine Kufa Iraq; ^3^ Research Unit Najaf Health Bureau Ministry of Health Najaf Iraq

## CONFLICT OF INTEREST

The authors declare that they have no conflict of interest.

Dear Editor,

We thank Dr. Jiang F for the positive feedback, and we appreciate the interest in our article describing electrocardiographic markers of increased risk of sudden cardiac death in patients with COVID‐19 pneumonia and for taking the time to express his comments (Alareedh et al., 2021).

In his letter to the editor, Dr. Jiang F discussed the association of myocardial or cardiac injury related to COVID‐19 infection with ECG changes and adverse clinical outcome. We also thought that the presence of cardiac injury related to COVID‐19 infection is associated with significant alterations in cardiac conduction and/or repolarization properties, which can predispose to malignant ventricular arrhythmias and sudden cardiac death (Wang et al., 2020).

Different mechanisms have been suggested for cardiac injury related to COVID‐19 infection, including myocardial expression of ACE2 on their surface leading to direct myocardial injury, hypoxia, alterations in ion channels, and cytokines storm. All these potential mechanisms potentiate myocardial injury associated with COVID‐19 infection that might be detected as various changes in surface ECG (Mehraeen et al., 2020; Wang et al., 2020).

However, the diagnosis of myocarditis is difficult to confirm consistently as diagnosis of possible myocarditis associated with COVID‐19 is based on clinical presentation and results of noninvasive imaging without myocardial biopsy or autopsy examinations (Bonow et al., 2020).

In our study, we did not assess the possibility of cardiac involvement by echocardiography or serum markers of cardiac damage, such as troponin or D‐dimer, to exclude myocardial injury as we mentioned in the limitation section of our article.

In his letter, Dr. Jiang F mentioned the importance of prethrombotic state and thrombotic state associated with COVID‐19 infection, which may lead to pulmonary embolism or coronary thrombosis even in the absence of cardiac risk factors. We agree with the Dr. that prethrombotic state and thrombotic state could potentially play a role in ECG changes or adverse clinical outcome associated with COVID‐19 infection. The possible underlying causes of ECG changes, such as pulmonary embolism or coronary disease, were beyond the scope of our study aims because of logistic limitations posed by isolation wards and shortage of health resources, which limits the assessment of all possible confounding factors that might influence ECG changes as we mentioned in the limitation section of our article.

Finally, as the clinical course of COVID‐19 evolves rapidly, ECG provides a quick, simple, and effective assessment of the patient's prognosis, and ECG changes detected on admission may be used as a sixth vital sign with possible prognostic value (Elias et al., 2020).

The limitations of our study should be kept in mind while interpreting its results and it would be of particular interest to assess the underlying causes of ECG changes and to determine the prognostic value of these ECG changes in patients with COVID‐19 infection in follow‐up studies.

## Data Availability

The data that support the findings of this study are available from the corresponding author upon reasonable request.
